# Intestinal Osteosarcoma with Liver Metastasis in a Dog with a History of Recurrent Cotton-Based Toy Fragment Ingestion

**DOI:** 10.3390/vetsci11120632

**Published:** 2024-12-07

**Authors:** Andrada Negoescu, Claudiu Gal, Andrei Mihaila, Constantin Mihaila, Cornel Cătoi, Marian Taulescu

**Affiliations:** 1 Department of Anatomic Pathology, Faculty of Veterinary Medicine, 400372 Cluj-Napoca, Romania; cornel.catoi@usamvcluj.ro (C.C.); marian.taulescu@usamvcluj.ro (M.T.); 2 Department of Veterinary Pathology, Synevovet, 81 Pache Protopopescu, 021408 Bucharest, Romania; gal.claudiu@gmail.com; 3 Andivet Hospital, 330004 Deva, Romania; mihaila19@gmail.com (A.M.); dr.mihaila@yahoo.com (C.M.)

**Keywords:** intestine, extraskeletal osteosarcoma, immunohistochemistry, cotton fibers, dog

## Abstract

Canine extraskeletal osteosarcomas are mesenchymal, osteoid producing tumors that can arise in soft tissues without initial involvement of the bones. An 8-year-old intact male Beagle dog presented with anorexia, abdominal pain, intermittent vomiting and melena. The patient had a history of recurrent ingestion of cotton based-toy fragments, but no prior surgical procedures involving the abdominal cavity. During the exploratory laparotomy, a mass was identified in the jejunal wall. Surgical resection was performed, and tissue samples were collected for pathological examination. Histologically, the mass was diagnosed as osteoblastic osteosarcoma with fragments of cotton fiber material. The neoplastic cells were immunolabeled for vimentin and BMP-2, further supporting the morphological diagnosis. Seven months after the surgery, metastatic nodules were identified in the liver. The dog died ten months after intestinal mass resection. This case represents the first documented instance of metastatic intestinal osteosarcoma potentially caused by ingestion of cotton fiber material.

## Simple Summary

This study reports a rare case of metastatic intestinal extraskeletal osteosarcoma in a dog, with a potential etiology linked to ingestion of cotton fiber material. An 8-year-old male Beagle presented with gastrointestinal symptoms and a history of repeated episodes of ingestion of cotton-based toy fragments, without previous abdominal surgeries. Exploratory laparotomy revealed a jejunal mass, which was surgically excised and confirmed as an osteoblastic osteosarcoma with embedded cotton fiber fragments through histopathological and immunohistochemical examinations. Despite surgery, the dog developed liver metastases and died ten months later.

## 1. Introduction

Canine osteosarcoma (OSA) is a malignant tumor with mesenchymal origin, characterized by osteoblasts’ neoplastic proliferation with osteoid formation [1]. It primarily affects the appendicular and axial skeleton, frequently the distal radius and proximal humerous [2], with a higher prevalence in large breeds (e.g., Irish Wolfhounds, Scottish Deerhounds, Great Danes) [3]. Extraskeletal osteosarcoma (EOS), a rare variant of osteosarcoma, develops in various soft tissues rather than bones. EOS has previously been reported in the salivary glands, intestines, heart, mammary glands, skin, subcutaneous tissue, blood vessels, liver, and lungs [4,5]. Chronic irritation, caused by certain factors, such as keratin, persistent inflammation, or retained surgical materials (gossypiboma or textiloma), is thought to be a major contributing factor in the development of this lesion. The potential mechanisms related to tumorigenesis may include the following: reactive nitrogen and oxygen species produced by macrophages during chronic granulomatous inflammatory responses against to the foreign bodies, as well as growth factors and cytokines [6,7,8]. EOS carries a poor prognosis due to its tendency to metastasize, particularly to the lungs, and the limited effectiveness of chemotherapy [4]. Even though primary intestinal osteosarcoma has previously been reported in dogs, this is the first case of EOS consistently associated with repeated episodes ingestion of cotton fiber material.

## 2. Case Description

An 8-year-old, intact male Beagle was presented to the clinic for a secondary opinion assessment. The patient’s clinical history included a lack of appetite, intermittent vomiting, and bloody stool. The owner mentioned the dog used to ingest fragments of cotton toys and had no prior surgical history involving the abdominal cavity. Additionally, the patient had no history of other medical conditions, such as metabolic disorders, and its deworming was up to date. Physical examination revealed severe abdominal distention and pain and pale gingiva. Rectal examination showed dark stool with mucus. The heart rate (approximately 100 bpm) was within the normal range. Pulmonary auscultation and lymph node palpation revealed unremarkable changes. A presumptive diagnosis was intestinal obstruction caused by an intestinal intussusception or neoplastic mass.

**Figure 1:**
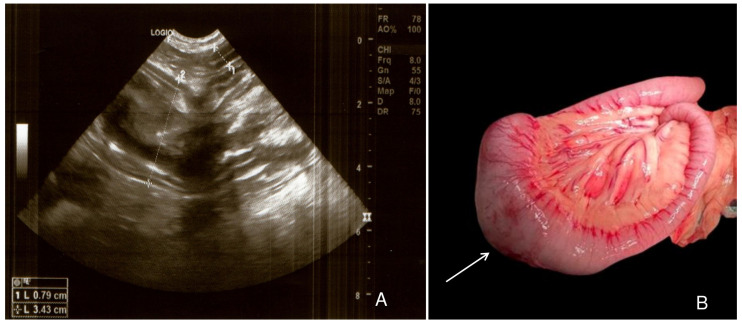
Ultrasound and macroscopical features of the canine intestinal osteosarcoma. The mass is characterized by a hypoechoic external region and an anechoic center (measured area 2); in the top right corner there is a normal intestinal loop (measured area 1); (**A**). Grossly, the intestinal wall is severely distended by a poorly delimited, infiltrative and dense mass (white arrow) (**B**).

An ultrasound scan was subsequently performed and an intestinal mass with a hypoechoic external area and an anechoic center, measuring 3.43 cm in diameter was identified (Figure 1 A). An exploratory laparotomy was decided upon in order to remove the intestinal mass. The blood count showed severe anemia (RBC: 2.06 × 10^12^/L with a normal range 5.5 × 10^12^/L to 8.5 × 10^12^/L, HCT: 18.25% normal range of 37% to 55%, HGB: 4.6 g/dL with a normal range 12 g/dL to 18 g/dL), with low total protein (3.6 g/dL with a normal range 5 g/dL–7.2 g/dL) and albumin (1.6 g/dL with a normal range 2.6 g/dL–4 g/dL) levels. Before the surgery, a transfusion of 1 unit of Dog Erythrocyte Antigen (DEA) negative blood was performed.

The protocol used for the anesthesia consisted of butorphanol 0.25 mg/kg, diazepam 0.5 mg/kg, ketamine 2 mg/kg, lidocaine 1 mg/kg, propofol 1 mg/kg, and for the maintenance of the anesthesia, isoflurane was used. The patient was positioned for surgery in the dorsal recumbency, and midline celiotomy was performed. During the examination of the abdominal cavity, a mass in the jejunum (Figure 1B) and a hematoma adjacent to the tumor were noted. Enterectomy with enteroanastomosis was performed using polydioxanone 2/0 and 3/0. A standard abdominal procedure was performed to close the abdominal cavity.

Post-surgery, the patient showed significant improvement and resumed eating. The dog remained hospitalized in the intensive care unit for 5 days, receiving intravenous fluids, antibiotics, pain medication, hemostatic agents, and anti-nausea medication. Tissue samples from the jejunal mass were fixed in 10% neutral buffered formalin and submitted for further investigation. The specimens were trimmed and routinely processed for microscopical evaluation. The paraffin-embedded samples were sectioned at a thickness of 2 µm and stained with Hematoxylin and Eosin (H&E) and Masson’s Trichrome (MT). Immunohistochemical analysis was performed using the following antibodies: vimentin (ready-to-use, V9, Leica Biosystems, Newcastle upon Tyne, UK) and bone morphogenetic protein 2 (BMP-2) polyclonal antibody (dilution 1:100, Invitrogen, Thermo Fisher Scientific). For vimentin, the staining was performed automatically using the Leica Bond-Max system, while for BMP-2 the procedure was conducted manually with a Dako EnVision Flex+ kit (Mouse, high pH), and the antigen retrieval was achieved using heat-induced epitope retrieval at pH 6. Whole slides images were acquired using a digital scanner (IntelliSite Ultra-Fast Scanner Philips, Best, The Netherlands) at the equivalence of 40 times magnification. The microscopical images were acquired using the Philips Image Management System (software version 3.3.7).

**Figure 2:**
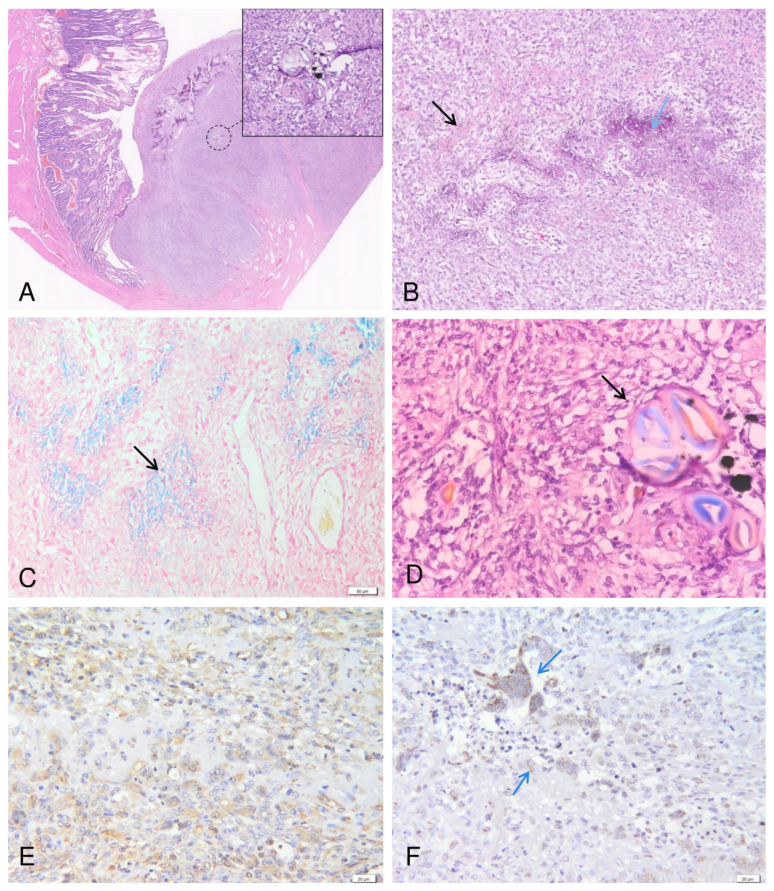
Photomicrographs of the canine intestinal osteosarcoma. The jejunum is transmurally infiltrated and replaced by a neoplastic mass containing cotton fiber fragments (the inset) (**A**). The neoplastic cells are polygonal to spindle, arranged in interwoven streams and bundles, associated with numerous islands of homogenous acidophilic extracellular matrix/osteoid (black arrow) partially mineralized (blue arrow) (**B**), confirmed by MT stain (black arrow) (**C**). The fragments of foreign material exhibit birefringence under polarized light (arrow) (**D**). Immunohistochemically, the neoplastic cells exhibit a strong cytoplasmatic expression for vimentin (**E**), and moderate cytoplasmatic expression for BMP 2 (blue arrows) (**F**).

Microscopically, the jejunum was transmurally infiltrated and replaced by an unencapsulated, poorly demarcated, ulcerated, and highly cellular neoplastic mass (Figure 2A). The neoplastic cells were arranged in interwoven streams, bundles, or surrounding a homogeneous pale eosinophilic material interpreted as osteoid (Figure 2B) and confirmed by MT stain (Figure 2C); in other areas of the tumor the osteoid islands were partially mineralized. The neoplastic cells were spindle to polyhedral, with indistinct cell borders, pale eosinophilic cytoplasm and round to oval nuclei with finely stippled chromatin, and a prominent magenta nucleolus. Moderate anisocytosis and anisokaryosis were observed, with occasional binucleated and multinucleated neoplastic cells. The number of mitoses ranged from 0 to 2 /high power field (14/2.37 mm^2^). Fiber-like material fragments displaying birefringence under polarized light (Figure 2D), interpreted as cotton fibers, were multifocally identified within the neoplastic tissue and surrounded by scattered neutrophils, macrophages, small lymphocytes and plasma cells.

Immunohistochemically, the neoplastic cells showed strong cytoplasmic expression for vimentin (Figure 2E) and low to moderate cytoplasmic labelling for BMP-2. Additionally, BMP-2 was strongly expressed in the neoplastic multinucleated cells and in those tumor cells adjacent to the foreign material (Figure 2F).

Based on morphological and immunohistochemical findings, a diagnosis of intestinal osteoblastic osteosarcoma was made.

Chemotherapy was declined by the owner due to financial constraints.

### Outcome

**Figure 3:**
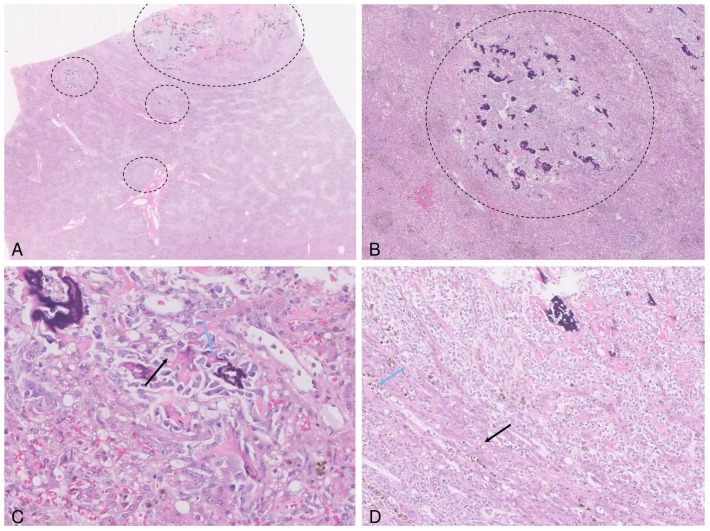
Photomicrographs of the metastatic osteosarcoma in the liver. The hepatic parenchyma is multifocally expanded and replaced by variably sized neoplastic nodules (delimited areas) (**A**,**B**). The tumor cells (black arrow) are intermingled with small islands of partially mineralized osteoid (blue arrow) (**C**). The adjacent parenchyma is compressed, contains small lipogranulomas (blue arrow) and the hepatocytes show vacuolar degeneration/lipidosis (black arrow) (**D**).

After the histopathological diagnosis (3 weeks later), a chest x-ray was performed, and no metastatic nodules were identified. Six months post-surgery, the patient presented again to the clinic with pale mucosae and severe hepatic injury (as indicated by biochemical markers). Ultrasound evaluation of the abdomen showed multiple hepatic nodules, involving all lobes and abdominal fluid. No other primary masses in other tissues (e.g., bones) or intestinal recurrence have been observed. A second surgical procedure was conducted, during which hepatic masses and hemoabdomen, secondary to severe necrosis of one of the nodules, were noted. Furthermore, tissue samples from hepatic nodules were collected for histopathological analysis. Microscopical examination of these biopsies confirmed the diagnosis of metastatic osteosarcoma to the liver (Figure 3A–D). The patient died three months later, without undergoing a postmortem examination.

## 3. Discussion

Osteosarcomas (OSAs) are malignant neoplasms originating from osteoblasts and possess the ability to synthesize osteoid. They are the most significant primary bone neoplasms, accounting for approximately 80% to 85% of such tumors. OSAs are frequently diagnosed in dogs, cats, and humans, as well as in rats, though they are rarely found in other species [9]. Extraskeletal OSAs in dogs are uncommon tumors, with documented occurrences in the mammary gland, subcutis, spleen, liver, gastrointestinal tract, and urogenital tract [6,7,8].

In the present study, we describe the morphological and immunohistochemical features of an extraskeletal intestinal osteosarcoma with liver metastases caused by frequent ingestion of textile fibers in a dog.

Intestinal OSA has previously been reported in four dogs with clinical history of vomiting, abdominal pain and effusions, anemia, weight loss, and decrease in appetite [10,11,12,13]. In these cases, the tumors were found in the small intestine (one case with duodenal location [13] and three cases with jejunal masses [10,11,12]) and these findings are in line with our reported data. The diagnosis of intestinal OSA is based on ultrasound examination and exploratory laparotomy, followed by pathological investigation.

Microscopically, OSAs are classified based on the quantity of osteoid produced and the morphological features of the neoplastic cells, including osteoblastic, fibroblastic, chondroblastic, telangiectatic, giant cell, poorly differentiated, myxoid, epithelioid and round cell types [14]. Herein, we describe an osteoblastic type of osteosarcoma. The neoplastic cells showed moderate to strong immunoexpression for both Vimentin and BMP2, these results being supported by previous investigations [15,16].

Due to their low incidence, the precise etiology of extraskeletal OSAs remains unclear. In certain instances, some of these tumors occurred secondary to local inflammatory reactions caused by foreign bodies. In dogs, there are few reports of extraskeletal osteosarcoma induced by various foreign bodies, in different anatomical regions (Table 1).

The mechanism involved in the development of foreign body-induced sarcoma remains unclear. However, potential factors that may contribute to tumorigenesis include the production of reactive nitrogen and oxygen species by macrophages during chronic granulomatous inflammatory responses against to the foreign bodies, observed in Trp53+/− mice with subcutaneous foreigner body implants in which there was an acceleration in the development of sarcomas [22]. Furthermore, elevated levels of interleukin-6 (IL-6), interleukin-8 (IL-18), interleukin-1ß (IL-1ß), Tumor Necrosis Factor-α (TNF-α), Transforming Growth Factor-ß (TGF-ß), chemokine (C-X-C motif) ligand 1 (CXCL1), chemokine (C-X-C motif), and ligand 2 (CXCL2) have been noted in tumors induced by foreign materials, suggesting their potential significant role in tumor development [23,24].

In one of the three reported cases, the intestinal osteosarcoma was induced by a retained surgical sponge after an elective ovariohysterectomy and involved only the intestinal serosa and outer muscle layer of the jejunal wall [10]. In our case, the mass affected all jejunal wall layers and we hypothesized that the ingested cotton fibers induced mucosal necrosis/ulceration, passed through the injured intestinal mucosa and entered in the intestinal wall, triggering a foreign body inflammatory reaction and resulting into osteosarcoma development. However, due to minimal inflammatory granulomatous reaction around the cotton fibers found at the time of pathological diagnosis, the term “gossybipoma-induced sarcoma” was not applied.

In humans, extraskeletal osteosarcomas account for approximately 1% of all soft tissue neoplasms and are associated with a poor survival rate. Regarding the survival rate of extraskeletal osteosarcoma, the prognosis is generally poor, with metastasis frequently occurring, particularly in the lungs via hematogenous spread [25].

In appendicular osteosarcoma, the primary treatment approach includes surgery (typically amputation), radiotherapy (used as a palliative measure), and chemotherapy with agents such as carboplatin, cisplatin, and doxorubicin [26]. The survival rate for dogs undergoing both surgery and chemotherapy generally extends up to one year, with occasional cases reaching survival up to two years [27,28]. Regarding the extraskeletal osteosarcoma induced by foreign bodies, a definitive survival rate or treatment efficacy remains unknown. This may be attributed to the limited number of reported cases, refusal of chemotherapy by pet owners, lack of follow-up evaluations, and early euthanasia of affected animals [4,29]. In our study, the patient survived approximately 10 months following the surgery, with liver metastasis detected six months post-diagnosis.

## 4. Conclusions

To our knowledge, this case represents the first documented instance of extraskeletal intestinal osteosarcoma induced by recurrent ingestion of cotton based-toy fragments.

## Figures and Tables

**Table 1:** Extraskeletal foreign body-induced osteosarcomas in dogs: anatomical location, causes, treatment and follow-up.

Age (Years)	Breed	Gender	Location	Cause	Treatment	Follow-up	Source
14	Miniature pinscher	M	subcutaneous (mandibular region)	salivary gland cyst (mucus accumulation in the mandible)	surgical resection no chemotherapy	NM	[17]
12	French bulldog	M	subcutaneous (mandibular region)	right salivary gland cyst	surgical resection no chemotherapy	PS days 204 metastasis in the lungs PS days 393 mass in the left eye PS days 451 patient died	[18]
10	Japanese Shiba Inu	F	mesentery	polyester and rayon surgical swab	surgical resection no chemotherapy	NM	[19]
8	Italian greyhound	F	abdominal cavity with adherences on the pancreas, stomach, jejunum, and cecum	cloth or surgical sponge	surgical resection piroxicam (0.3 mg/kg PO q 24 h) (administrated for a year)	No metastasis was identified a year PS	[1]
11	Labrador Retriever	F	medial aspect of the stifle	cotton gauze	surgical resection doxycycline (5 mg/kg PO BID) deracoxib (2 mg/kg PO SID)	3 months PS local recurrence (the dog was euthanized)	[20]
13	Cocker Spaniel mixed breed	F	subcutaneous	keratin from a benign hair follicle tumor	carboplatin (260 mg/m^2^ IV, q3 wk for 5 treatments) toceranib 2.6 mg/kg body weight, PO, given on a Monday, Wednesday, Friday weekly schedule	37 months PS lung mass 45.4 months PS dog was euthanized (unrelated disease)	[21]

NM—not mentioned; PO—per os; PS—post surgery; BID—bis in die (twice a day); SID—semel in die (once daily).

## Data Availability

The raw data supporting the conclusions of this article will be made available by the authors on request.
